# Diet-Related Quality of Life Reflects Psychological and Autonomic Burden in Patients with Dizziness and Balance Disorders: A Cross-Sectional Study

**DOI:** 10.3390/nu18132044

**Published:** 2026-06-23

**Authors:** Shinnosuke Asakura, Teru Kamogashira, Hideaki Funayama, Hibiki Yabe, Toshitaka Kataoka, Shizuka Shoji, Megumi Koizumi, Wakako Nakanishi, Shinichi Ishimoto

**Affiliations:** 1 Department of Clinical Examination, JR Tokyo General Hospital, Tokyo 151-8528, Japan; 2 Department of Otolaryngology, JR Tokyo General Hospital, Tokyo 151-8528, Japan; 3 Department of Otolaryngology and Head and Neck Surgery, Faculty of Medicine, University of Tokyo, Tokyo 113-8655, Japan

**Keywords:** diet-related quality of life, dizziness and balance disorders, psychological distress, autonomic dysfunction, migraine, quality of life

## Abstract

Background/Objectives: This study aimed to examine the associations between diet-related quality of life (DRQOL) and psychological distress, autonomic dysfunction, and migraine in patients with dizziness and balance disorders. Methods: In this retrospective cross-sectional study, 122 patients (56 men, 66 women; mean age 40.4 ± 12.8 years, minimum 14, maximum 65) from the vertigo outpatient clinic at JR Tokyo General Hospital completed self-reported questionnaires. These included the DRQOL scale, Dizziness Handicap Inventory (DHI), Hospital Anxiety and Depression Scale (HADS), Self-rating Depression Scale (SDS), Orthostatic Dysregulation (OD) checklist, and migraine assessments (POUNDing [Pulsating, duration of 4–72 h, Unilateral, Nausea, Disabling], MIDAS, migraine screener). Correlational analyses, group comparisons, and receiver operating characteristic (ROC) analyses were conducted. Results: Higher DRQOL scores indicate poorer DRQOL. DRQOL scores showed positive correlations with psychological distress (SDS: ρ = 0.57; HADS-A: ρ = 0.50; HADS-D: ρ = 0.53; all *p* < 0.001) and OD severity (ρ = 0.50, *p* < 0.001) but not with age, DHI, or individual migraine indices. Migraine screener-positive patients had significantly higher DRQOL scores (*p* < 0.01). DRQOL alone showed modest ability to discriminate migraine screener-positive from migraine screener-negative patients (AUC = 0.65); discrimination improved to an AUC of 0.77 in a multivariable model that also included age and sex. Conclusions: DRQOL appears to capture psychological and autonomic symptom burden rather than vestibular or headache severity, suggesting that it may serve as a complementary, patient-centered metric that adds a multidimensional perspective to conventional vestibular and headache assessments.

## 1. Introduction

Vertigo, dizziness, and balance disorders are highly prevalent conditions that substantially impair daily functioning and quality of life (QOL), and are frequently associated with psychological distress, reduced balance confidence, and cognitive burden [1,2]. Migraine is widely recognized as one of the major conditions associated with dizziness and unsteadiness, making its identification and management particularly important in clinical practice [3,4]. Additionally, autonomic dysfunction, including orthostatic dysregulation (OD), frequently coexists with vestibular symptoms and migraine, contributing to the complexity of symptom presentation.

Dietary factors have long been recognized as potential triggers of migraine attacks. Previous studies have mainly focused on the role of specific foods or irregular eating patterns, such as alcohol, caffeine, chocolate, and meal skipping, in relation to migraine exacerbation [5,6]. Although the overall evidence remains inconsistent, dietary modification may reduce migraine frequency, severity, and disability in some patients [5,7]. More recently, research has shifted from individual triggers to broader dietary patterns, with higher overall diet quality inversely associated with migraine outcomes [6]. However, these approaches have primarily focused on nutritional composition rather than the experiential aspects of eating.

While nutritional content is important, increasing attention has been directed toward diet-related quality of life (DRQOL), a multidimensional construct encompassing satisfaction, enjoyment, sensory aspects, and social dimensions of eating [8,9,10,11]. DRQOL has been shown to be relevant in various chronic conditions, including multiple sclerosis and osteoarthritis, highlighting its potential importance as a patient-centered outcome measure [12,13]. Although dizziness-specific patient-reported outcomes, such as the Dizziness Handicap Inventory (DHI), quantify dizziness-related handicap, they do not capture diet-related experiences, including food avoidance, loss of enjoyment, and social restrictions surrounding eating, which may be clinically relevant in patients with dizziness and migraine. Despite accumulating evidence regarding dietary factors in migraine, the comprehensive assessment of DRQOL in patients with dizziness and balance disorders has not been sufficiently explored [14,15]. Patients with dizziness and migraine may experience nausea, altered eating behaviors, or concerns regarding symptom exacerbation after meals, which can adversely affect DRQOL [3,4].

Gastrointestinal and autonomic symptoms accompanying vestibular disorders may also contribute to impaired sensory and emotional aspects of eating [1,5,16]. These interactions may be understood within a broader gut–brain–vestibular framework [17]. Emerging evidence suggests that brainstem and central autonomic networks integrate vestibular, visceral, autonomic, and nociceptive signals, contributing to symptom generation in both migraine and dizziness [3]. Altered interoceptive processing may further influence how internal bodily sensations are perceived and interpreted, potentially affecting dietary experiences and DRQOL [18]. However, the contribution of these mechanisms to diet-related outcomes in patients with dizziness and balance disorders remains to be clarified [19].

Although DRQOL has been investigated in several chronic disease populations [20,21] and is increasingly recognized as an important patient-reported outcome, its application in neuro-otological practice has received relatively little attention. Therefore, the present study was designed as an exploratory investigation to characterize the potential relevance of DRQOL in patients with dizziness and balance disorders. In particular, the relationships between DRQOL and migraine comorbidity, psychological factors such as anxiety and depression, and autonomic symptoms remain unclear [22]. Given that dizziness, migraine, psychological distress, and autonomic dysfunction frequently coexist and may influence daily dietary experiences, a more holistic evaluation of DRQOL may provide clinically meaningful insights beyond conventional dietary assessments.

The present study aimed to comprehensively examine the associations between DRQOL and migraine-related indicators, psychological factors, and autonomic symptoms in patients with dizziness and balance disorders.

## 2. Materials and Methods

This was a retrospective study conducted at a single institution. Patients under 65 years referred to the Vertigo Outpatient Clinic at JR Tokyo General Hospital between January 2023 and November 2025 were included in this study. Age eligibility was determined using unrounded age values; ages reported in the manuscript were rounded to the nearest year. The clinical study was approved by the regional ethical standards committee of JR Tokyo General Hospital (approval code: R06-21; approval date: 28 January 2025). The information for this study was disclosed, and the participants could choose to opt out. An opt-out informed consent protocol was utilized to collect participant data for research purposes. Diagnoses were made in accordance with established diagnostic criteria, including the Bárány Society criteria where applicable (for Ménière’s disease and vestibular migraine). Data on scores of questionnaires regarding psychological symptoms were collected. Questionnaires administered included Dizziness Handicap Inventory (DHI) [23], Hospital Anxiety and Depression Scale (HADS) [24], Self-rating Depression Scale (SDS) [25], OD checklist score (a symptom-burden measure, distinct from a diagnosis of OD) [16,26,27], POUNDing (Pulsating, duration of 4–72 h, Unilateral, Nausea, Disabling) [28], Migraine Disability Assessment (MIDAS) Grade (I–IV) [29], 4-item migraine screener (headache exacerbation in daily performance, nausea, light-sensitivity, and hypersensitivity to odors) [30]. In addition, participants reported the number of headache days over the past 3 months (range, 0–90) and headache intensity of the most recent episode, assessed using an 11-point numeric rating scale (NRS; 0 = no headache, 10 = worst imaginable headache). The DRQOL questionnaire consists of the following 18 items [(1) Did you find your meal delicious? (2) Did you feel satisfied after your meal? (3) Did you enjoy the colors and presentation of your meal? (4) Did you enjoy the aroma of your meal? (5) Did you eat your meal at the appropriate temperature (i.e., hot foods served hot and cold foods served cold)? (6) Did you eat your meal with a pleasant texture in a desirable state? (7) Did you eat the amount you wanted? (8) Did you consume a variety of foods? (9) Did you eat foods you like? (10) Did you have your meal with family or friends? (11) Did you eat in a relaxed atmosphere? (12) Did you say “delicious” while eating your meal? (13) Did you use your preferred tableware during your meal? (14) Did you enjoy your meal? (15) Did you feel full after your meal? (16) Do you still enjoy the taste of your hometown or home-cooked meals? (17) Do you often eat seasonal foods? (18) Do you regularly attend seasonal events or traditions (e.g., New Year, Hina Matsuri, Moon Viewing)?], with respondents selecting from five options [1: Always, 2: Almost always, 3: Sometimes, 4: Rarely, and 5: Never], and the total score is calculated (maximum 90, minimum 18) [19,31]. Although this instrument was originally developed and validated in community-dwelling older adults, it assesses broad domains of DRQOL, including meal satisfaction, enjoyment, sensory experiences, and social aspects of eating. Given the lack of established DRQOL measures specifically for patients with dizziness and balance disorders, we applied this instrument exploratorily to a younger and clinically diverse cohort to evaluate its potential utility in this population. Higher scores indicate poorer DRQOL. All of these evaluations were conducted using self-administered questionnaires.

Microsoft Excel 365 for enterprise (version 2511, Microsoft, Redmond, WA, USA) was used for processing data. Statistical analyses were performed using R version 4.5.2 software (R Core Team; R Foundation for Statistical Computing, Vienna, Austria, 2025) [32] with the following packages (version): tableone (0.13.2), pROC (1.19.0.1), readxl (1.4.5), corrplot (0.95), psych (2.6.5), Hmisc (5.2-5), devtools (2.4.6), ggplot2 (4.0.1), diptest (0.77-2), car (3.1-5), boot (1.3-32), epiDisplay (3.7.0.0), tidyr (1.3.2), dplyr (1.2.1), and Rmisc (1.5.1) (Appendix A). Data are expressed as mean ± standard deviation (SD). Continuous variables were compared using the Mann–Whitney U test because of non-normal distributions, and categorical variables were compared using Fisher’s exact test. Associations between questionnaire scores were assessed using Spearman’s rank correlation coefficients. For key correlations, 95% confidence intervals were estimated using bootstrap resampling (1000 iterations). To account for multiple comparisons, *p*-values from Spearman correlation analyses were adjusted using the Holm method. Statistical significance was determined based on the adjusted *p*-values. Multivariable logistic regression analysis was conducted to assess the combined discriminative ability of age, sex, and DRQOL for positive migraine screener. Receiver operating characteristic (ROC) curve analysis was used to evaluate the discriminative ability of DRQOL and to determine the optimal cutoff value using the Youden index. The area under the ROC curve (AUC) and its 95% confidence interval were calculated using the DeLong method. To assess the modality of the DRQOL score distribution, Hartigan’s dip test for unimodality versus multimodality was applied. A two-sided *p* value < 0.05 was considered statistically significant.

## 3. Results

**Figure 1:**
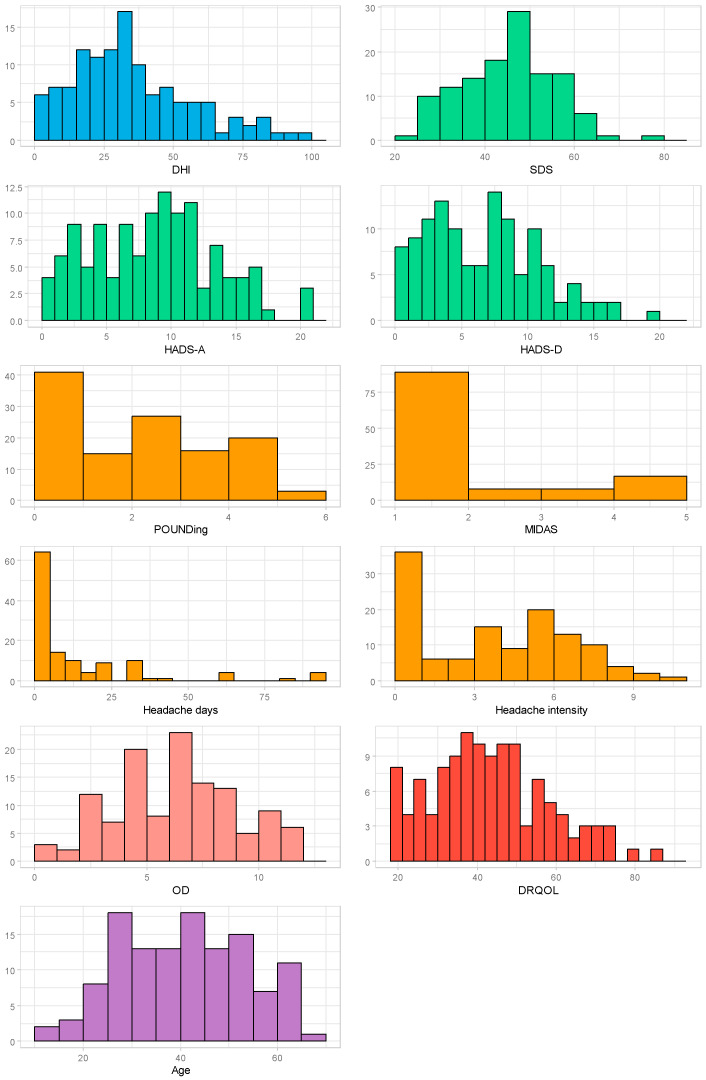
Distribution of questionnaire scores and headache-related variables. DHI, SDS, HADS (HADS-A and HADS-D), POUNDing, MIDAS, headache days, headache intensity, OD checklist score, and DRQOL score are shown. *Y*-axis: number of cases. Abbreviations are defined in the Abbreviations section.

A total of 156 patients were initially considered. After excluding 31 patients aged 65 years or older, 1 patient with no completed questionnaires, 1 patient with missing migraine-related questionnaire data, and 1 patient with missing HADS data, 122 patients (56 men and 66 women) were included in the study, with a mean age of 40.4 ± 12.8 years. The distribution of diagnoses was as follows: benign paroxysmal positional vertigo (BPPV) in 18 patients, definite Ménière’s disease (MD) in 11 patients, probable MD in 12 patients, sudden sensorineural hearing loss with vertigo in 1 patient, vestibular neuritis in 5 patients, peripheral vestibular disorder in 3 patients, idiopathic bilateral vestibulopathy (IBV) in 2 patients, congenital nystagmus in 2 patients, tension-type headache in 1 patient, migraine in 13 patients, vestibular migraine in 7 patients, psychogenic vertigo (functional dizziness) in 30 patients, and OD in 17 patients. Figure 1 presents the distributions of questionnaire scores and headache-related variables, including DHI, SDS, HADS (HADS-A and HADS-D), POUNDing, MIDAS, headache days, headache intensity, OD checklist score, and DRQOL score. The mean ± SD values were as follows: DHI, 34.7 ± 21.6; SDS, 44.8 ± 10.1; HADS-A, 8.2 ± 4.8; HADS-D, 6.2 ± 4.3; POUNDing, 1.7 ± 1.6; MIDAS, 1.6 ± 1.1; headache days, 12.6 ± 20.7; headache intensity, 3.3 ± 2.8; OD checklist score, 5.8 ± 2.7; and DRQOL score, 42.7 ± 15.0. The DRQOL scores showed a wide, approximately unimodal distribution, ranging from about 18 to 90 (mean ± SD: 42.7 ± 15.0), with Hartigan’s dip test confirming the absence of multimodality (D = 0.02, *p* = 0.97). This wide distribution suggests substantial interindividual variability in DRQOL among patients with dizziness and balance disorders.

**Figure 2:**
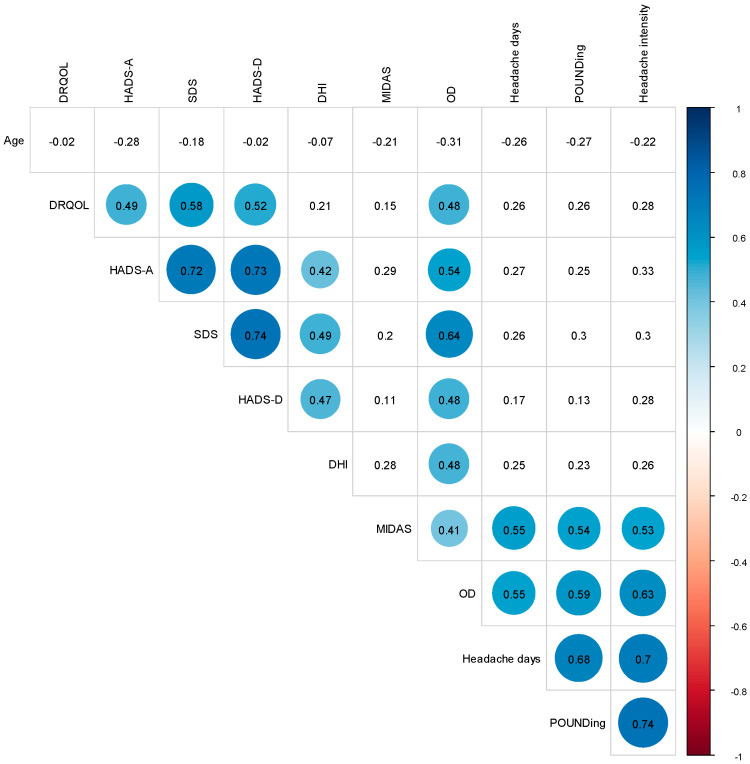
Correlation coefficients between questionnaire scores and headache-related variables. Numbers within the cells indicate Spearman’s correlation coefficients. Filled circles indicate correlations significant after Holm correction for multiple testing (55 comparisons; adjusted *p* < 0.001); correlations not meeting this threshold are shown as numbers without circles. The more stringent adjusted *p* < 0.001 threshold was adopted to improve figure readability. Circle color denotes the direction of the correlation, and color intensity reflects the magnitude of the correlation coefficient, as shown in the vertical color bar on the right. Correlation coefficients shown in the figure were obtained from the complete-case analysis; confidence intervals reported in the main text were estimated using bootstrap resampling. Abbreviations are defined in the Abbreviations section.

Correlations among questionnaire scores and headache-related variables are shown in Figure 2. DRQOL score demonstrated selective associations with psychological and autonomic measures while there were no significant correlations with age or dizziness-related disability. Specifically, DRQOL score was significantly associated with depressive symptoms assessed by SDS (ρ = 0.57, 95% CI 0.42–0.68, adjusted *p* < 0.001), HADS-A (ρ = 0.50, 95% CI 0.36–0.63, adjusted *p* < 0.001), and HADS-D (ρ = 0.53, 95% CI 0.41–0.65, adjusted *p* < 0.001). In addition, a significant association was observed between DRQOL score and OD checklist score (ρ = 0.50, 95% CI 0.37–0.61, adjusted *p* < 0.001). These findings indicate that higher DRQOL scores were associated with a higher burden of psychological and autonomic symptoms.

To assess the potential influence of psychogenic dizziness, a sensitivity analysis was performed after excluding patients with psychogenic dizziness, leaving 92 patients for analysis. The associations between DRQOL score and SDS (ρ = 0.63, adjusted *p* < 0.001), HADS-A (ρ = 0.46, adjusted *p* < 0.001), HADS-D (ρ = 0.48, adjusted *p* < 0.001), and OD checklist score (ρ = 0.52, adjusted *p* < 0.001) remained significant. In addition, significant correlations were observed between DRQOL score and POUNDing score (ρ = 0.34, adjusted *p* = 0.02) as well as headache intensity (ρ = 0.33, adjusted *p* = 0.03). In the non-psychogenic subgroup, weak but significant associations with migraine-related indices (POUNDing score and headache intensity) additionally emerged, suggesting that inclusion of patients with psychogenic dizziness may have attenuated these relationships. Overall, the pattern of associations was largely preserved after exclusion of patients with psychogenic dizziness.

In contrast, no significant associations were observed between DRQOL score and age (ρ = −0.01, adjusted *p* > 0.05), DHI (ρ = 0.21, adjusted *p* > 0.05), or migraine-related indices, including POUNDing, MIDAS, headache days over the past 3 months, and headache intensity of the most recent headache (all adjusted *p* > 0.05). These findings suggest that DRQOL score may be more closely related to psychological distress and autonomic symptom burden rather than headache frequency or intensity itself.

Importantly, although DRQOL score was not significantly correlated with DHI, both DRQOL score and DHI showed significant associations with psychological distress measures, suggesting that DRQOL and dizziness-related handicap may reflect distinct but partially overlapping psychosomatic dimensions.

In addition to DRQOL score, correlations were observed among psychological measures, including SDS with HADS-A (ρ = 0.72, adjusted *p* < 0.001) and HADS-D (ρ = 0.74, adjusted *p* < 0.001), as well as between HADS-A and HADS-D (ρ = 0.73, adjusted *p* < 0.001). Migraine-related measures also showed robust interrelationships, with POUNDing score being significantly correlated with MIDAS, headache days, and headache intensity (all adjusted *p* < 0.001).

Notably, the OD checklist score showed significant correlations with a wide range of questionnaire scores and headache-related measures, including DHI, SDS, HADS-A, HADS-D, DRQOL, and multiple migraine-related indices, suggesting that OD checklist score may be associated with a broader symptom burden encompassing vestibular, psychological, autonomic, and headache-related domains. Among the assessed measures, OD checklist score demonstrated the broadest pattern of significant associations, suggesting its potential role as a global indicator of multisystem symptom burden in patients with dizziness and balance disorders.

Table 1 shows comparisons between patients stratified by migraine screener status. The migraine screener-positive group was significantly younger and included a lower proportion of males than the migraine screener-negative group. Patients who screened positive for migraine exhibited significantly higher scores across all vestibular, psychological, and headache-related measures, including DHI, SDS, HADS-A, HADS-D, POUNDing, MIDAS, headache days, headache intensity, and OD checklist score (all *p* < 0.05). In addition, DRQOL scores were significantly higher in the migraine screener-positive group, indicating poorer DRQOL, with a magnitude comparable to that observed for other psychological and symptom-related measures.

Although multiple questionnaire measures differed significantly between migraine screener-positive and migraine screener-negative groups, ROC analysis focused on DRQOL because it is the comprehensive, patient-centered outcome of primary interest in this study. ROC curve analysis showed an area under the curve (AUC) of 0.65 (95% CI, 0.55–0.75, DeLong method), indicating modest discriminative ability. The optimal DRQOL cutoff was 46.5, with sensitivity of 0.56 and specificity of 0.73. This analysis was performed in an exploratory manner to evaluate the potential discriminative value of DRQOL as a summary measure. Given that the migraine screener-positive group was significantly younger and included a lower proportion of males (Table 1), we performed multivariable logistic regression analysis incorporating age, sex, and DRQOL as predictors of positive migraine screener to assess their combined discriminative capacity. ROC curve analysis of the multivariable model showed improved performance compared to DRQOL alone, with an AUC of 0.77 (95% CI, 0.69–0.86, DeLong method). The improvement in AUC was statistically significant by DeLong’s test (*p* = 0.02) and was confirmed by bootstrap testing with 1000 resamples (*p* = 0.02). All predictors (DRQOL, sex, and age) remained significant (*p* < 0.05). The model demonstrated a sensitivity of 0.74 and specificity of 0.75. The adjusted odds ratios (95% CI) were as follows: DRQOL, 1.03 (1.01–1.07); sex, 0.30 (0.12–0.69); and age, 0.96 (0.93–0.99). Variance inflation factors (VIF) indicated minimal multicollinearity among predictors: DRQOL, 1.01; sex, 1.00; and age, 1.01.

Because psychological distress and autonomic symptom burden were significantly associated with both DRQOL and migraine screener status, an exploratory multivariable logistic regression analysis was additionally performed including HADS-A, HADS-D, SDS, and OD checklist scores together with age, sex, and DRQOL. In this model, DRQOL was not independently associated with migraine screener positivity (adjusted OR, 1.00; 95% CI, 0.96–1.04; *p* = 0.985). Neither HADS-A (adjusted OR, 1.15; 95% CI, 0.98–1.38; *p* = 0.100), HADS-D (adjusted OR, 0.92; 95% CI, 0.75–1.12; *p* = 0.393), nor SDS (adjusted OR, 0.97; 95% CI, 0.88–1.07; *p* = 0.597) was independently associated with migraine screener positivity. In contrast, OD checklist score remained significantly associated with migraine screener positivity (adjusted OR, 1.64; 95% CI, 1.26–2.26; *p* < 0.001), while male sex remained associated with lower odds of migraine screener positivity (adjusted OR, 0.35; 95% CI, 0.13–0.89; *p* = 0.032). The expanded model demonstrated good discriminative performance, with an AUC of 0.858 (95% CI, 0.791–0.925, DeLong method). VIFs were low for all predictors (DRQOL, 1.53; sex, 1.02; age, 1.17; HADS-A, 2.55; HADS-D, 2.91; SDS, 3.51; and OD, 1.80), indicating no evidence of problematic multicollinearity.

## 4. Discussion

### 4.1. Association of DRQOL with Psychological Distress and Autonomic Symptom Burden

In this study, DRQOL scores among patients with dizziness and balance disorders were significantly associated with psychological distress and autonomic symptom burden, but not with self-reported measures of dizziness-related disability or migraine indices. The positive correlations observed between DRQOL score and measures of anxiety, depression, and OD imply that greater psychological and autonomic burden is linked to poorer dietary well-being. In contrast, DRQOL score showed no significant association with age, DHI, or headache frequency and intensity.

Interestingly, patients who screened positive for migraine reported significantly higher DRQOL scores than those who did not, and ROC analysis revealed only modest discriminative ability of DRQOL score for identifying positive migraine screener status (AUC = 0.65). Importantly, a positive migraine screener does not constitute a clinical diagnosis of migraine or vestibular migraine, and therefore, these findings should not be interpreted as evidence that DRQOL can identify migraine disorders. The optimal cutoff value of 46.5 should likewise be considered exploratory and hypothesis-generating. External validation in independent cohorts and studies using established diagnostic criteria for migraine and vestibular migraine is required before any potential clinical application can be considered. Nevertheless, the observed association suggests that impaired DRQOL may be more common among patients reporting migraine-related symptoms [3,4]. However, the exploratory multivariable analysis suggested that this association should be interpreted with caution. Although DRQOL remained independently associated with migraine screener positivity after adjustment for age and sex, the association was no longer significant after additional adjustment for psychological distress (HADS-A, HADS-D, and SDS) and autonomic symptom burden (OD checklist score). In contrast, OD symptom burden remained independently associated with migraine screener positivity, whereas neither HADS-A, HADS-D, nor SDS reached statistical significance. These findings suggest that the observed relationship between impaired DRQOL and migraine may largely reflect underlying autonomic symptom burden rather than an independent effect of DRQOL itself. Accordingly, DRQOL may function primarily as a patient-centered indicator of overall symptom burden in patients with dizziness and balance disorders.

DRQOL may vary along a continuum of symptom burden, reflecting substantial interindividual variability rather than discrete clinical phenotypes; these interpretations should be considered hypothesis-generating and warrant prospective validation. Patients with low and high DRQOL scores may differ substantially in their dietary experiences and overall symptom burden. However, the specific mechanisms underlying these differences remain unclear because behaviors and experiences such as food avoidance, nausea, appetite changes, and eating-related anxiety were not directly assessed in the present study. These observations should therefore be considered hypothesis-generating and warrant prospective investigation [1,2].

The absence of a significant correlation between DRQOL score and DHI (ρ = 0.21, adjusted *p* > 0.05) despite both scales being associated with psychological distress suggests that they capture distinct domains of patient experience. While DHI primarily reflects functional limitations due to vestibular dysfunction, DRQOL appears to tap into psychosomatic aspects of eating, such as food-related anxiety, altered appetite, and diminished enjoyment of meals. This distinction underscores the value of DRQOL score as a non-redundant, patient-centered outcome that complements conventional vestibular assessments.

Although migraine screener-positive patients reported higher headache intensity than migraine screener-negative patients, headache intensity itself was not significantly correlated with DRQOL score after correction for multiple comparisons. This pattern suggests that DRQOL may be influenced by broader psychological and somatic factors beyond headache pain alone. However, specific contributors such as nausea, dietary restrictions, and eating-related anxiety were not directly assessed and require further investigation. The difference in DRQOL score may therefore reflect the cumulative impact of multiple migraine-related factors rather than pain severity in isolation.

OD demonstrated particularly broad associations across vestibular, psychological, autonomic, headache-related, and diet-related domains. Its correlation with DRQOL (ρ = 0.50, adjusted *p* < 0.001) likely stems from shared pathophysiological mechanisms, including autonomic dysfunction and altered cerebrovascular regulation. Patients with prominent orthostatic symptoms may experience gastrointestinal and eating-related symptoms that could adversely affect DRQOL; however, these factors were not directly measured in the present study. These findings highlight the importance of evaluating and managing orthostatic symptoms in patients with dizziness who also report dietary concerns. The OD checklist was originally developed for Japanese adolescents; therefore, its application to adults in the present study should be considered exploratory and requires further validation.

### 4.2. Interpretation of DRQOL as a Patient-Centered Outcome Measure

These findings were derived from a questionnaire-based analytical approach emphasizing dimensional symptom severity rather than categorical diagnoses. Although all participants were diagnosed using established criteria, our analyses focused on associations among self-reported symptoms across psychological, autonomic, vestibular, and dietary domains. Consequently, the observed relationships reflect the co-occurrence of subjective symptom burden within individuals seeking care for dizziness, rather than differences between discrete diagnostic entities. This dimensional framework can be particularly suitable for DRQOL, which captures experiential aspects of eating that are likely more closely related to psychological and somatic distress than to specific disease diagnoses.

### 4.3. Diet-Related Quality of Life and Potential Neurobiological Mechanisms

This study extends prior research on diet and migraine by shifting focus from specific food triggers or nutritional intake to the experiential dimensions of eating. Previous work has emphasized associations between migraine and individual dietary components (e.g., alcohol, caffeine, tyramine), irregular meal patterns, or specific dietary regimens (e.g., ketogenic, low-glycemic, Mediterranean diets) [5,7]. Although evidence remains limited, certain diets and food items may trigger migraine attacks in some individuals, and dietary modification has been reported to reduce attack duration, frequency, severity, and medication use [7]. Specific dietary factors, including alcohol, caffeine, chocolate, monosodium glutamate, nitrates, and tyramine, have been implicated as triggers in selected populations, while dietary interventions such as low-glycemic index diets, ketogenic diets, omega-3 supplementation, and Mediterranean dietary patterns have shown potential benefits [33]. In addition, a large-scale genome-wide association study using Mendelian randomization reported that intake of coffee, cheese, oily fish, alcohol (red wine), raw vegetables, muesli, and wholemeal/wholegrain bread was associated with a decreased risk of migraine, whereas consumption of white bread, cornflakes/frosties, and poultry was positively associated with migraine risk; additionally, intake of white bread, wholemeal/wholegrain bread, muesli, alcohol (red wine), cheese, and oily fish was linked to a higher risk of insomnia and major depressive disorder [34]. Dietary diversity has been linked to migraine burden, with individuals with less diverse diets tending to report higher frequency of migraine attacks [35]. Irregular meals and meal skipping have also been associated with increased migraine frequency [36]. Moreover, overarching dietary patterns such as the Mediterranean, Dietary Approaches to Stop Hypertension (DASH), Mediterranean-DASH Intervention for Neurodegenerative Delay (MIND), or low-glycemic diets are being studied for their potential to mitigate migraine symptoms via mechanisms including neuroinflammation, oxidative stress, regulation of vascular tone, and modulation of neuropeptides such as calcitonin gene-related peptide (CGRP), a key neuropeptide implicated in migraine pathophysiology and a major therapeutic target [37]. These dietary effects may be mediated, at least in part, through reductions in systemic inflammation and oxidative stress, as well as modulation of trigeminovascular signaling pathways involving CGRP [38,39]. Although inconsistency is present in the literature and no consensus exists, the available data are promising in supporting beneficial dietary interventions for some migraine patients [5]. In a study assessing diet quality using dietary intake, higher diet quality was inversely associated with migraine frequency and migraine-related disability; additionally, higher diet quality was linked to reduced migraine severity, suggesting that improved diet quality may be associated with favorable migraine outcomes, including lower headache frequency, reduced severity, and decreased migraine-related disability [6]. However, these approaches often overlook the subjective, qualitative aspects of eating.

These associations may also be interpreted within a broader neurobiological framework of gut–brain–vestibular interactions. Brainstem and central autonomic networks involved in migraine are known to integrate vestibular, visceral, autonomic, and nociceptive signals, thereby linking dietary experiences with dizziness, autonomic symptoms, and headache-related processes. Interoceptive mechanisms, which contribute to the perception and interpretation of internal bodily states, may be particularly relevant in this context. Altered interoceptive processing has been implicated in both migraine and functional somatic symptoms and may amplify the subjective impact of gastrointestinal discomfort, autonomic symptoms, and food-related experiences on QOL. Within this framework, impaired DRQOL may reflect not only dietary behaviors themselves but also altered integration of visceral and autonomic signals. The strong associations observed between DRQOL, psychological distress, and orthostatic symptom burden in the present study are consistent with this interpretation, although the underlying mechanisms remain speculative and require further investigation [40,41].

By contrast, DRQOL encompasses satisfaction, sensory pleasure, social engagement, and emotional well-being related to food, dimensions that are increasingly recognized as integral to overall QOL [11,42]. This approach reflects a broader perspective in which eating is viewed not merely as a means of obtaining nutrients but as an experiential process that contributes substantially to QOL [8,9,10,43]. Accumulating evidence suggests that psychological and cognitive factors play a central role in shaping eating behavior. Experimental studies have demonstrated that remembered enjoyment of food can influence subsequent food choice and intake [44], and that figurative or evocative food descriptions can enhance anticipated enjoyment and preference for healthier options [45]. These findings support the relevance of evaluating DRQOL as an experiential and psychological construct, rather than focusing solely on nutritional adequacy.

From a clinical standpoint, disease-specific instruments assessing DRQOL have been developed in other medical fields. For example, the Esophago-Gastric surgery and Quality of Dietary Life (EGQ-D), designed for patients undergoing esophagectomy or gastrectomy, has demonstrated good content and psychometric validity, supporting its utility as a disease-specific QOL measure [46]. Such instruments illustrate how DRQOL measures can capture aspects of daily life not adequately reflected by conventional clinical or nutritional indices. Although DRQOL has been examined in various chronic conditions, including multiple sclerosis [12] and osteoarthritis [13], a comprehensive assessment of DRQOL in patients with dizziness and balance disorders remains limited. The present findings suggest that DRQOL may be particularly relevant in relation to migraine comorbidity and psychological factors such as anxiety, depression, and somatization.

Furthermore, eating pleasure has been conceptualized as comprising multiple dimensions, including visceral satisfaction related to hunger alleviation and epicurean pleasure derived from sensory and aesthetic appreciation of food. Prior work has shown that higher epicurean tendencies are associated with greater well-being without adverse effects on body mass index, suggesting that qualitative aspects of eating experiences may contribute positively to overall QOL [47]. These observations are consistent with the present findings and support the hypothesis that DRQOL may reflect qualitative and psychological dimensions of dietary life. However, the specific pathways linking DRQOL with migraine-related symptoms and psychological distress remain to be determined.

### 4.4. Clinical Implications

The intercorrelations among psychological measures (SDS, HADS-A, and HADS-D; all ρ > 0.7) suggest substantial overlap in the constructs assessed by these instruments. From a practical standpoint, this raises the question of whether multiple psychological screening tools are necessary in routine clinical evaluation. However, each scale has distinct characteristics: HADS separately assesses anxiety and depression, while SDS provides a more comprehensive depressive symptom profile. The choice of screening instruments may depend on the specific clinical context and the primary outcomes of interest.

### 4.5. Limitations

Several limitations should be acknowledged. First, this was a single-center, cross-sectional study conducted in a real-world clinical setting, which limits generalizability and precludes causal inference. The study population was diagnostically heterogeneous, encompassing a wide range of dizziness-related conditions, including migraine-associated vertigo and psychogenic vertigo. While this heterogeneity reflects the complexity of patients typically encountered in routine dizziness clinics and supports the clinical relevance of DRQOL as a broadly applicable patient-reported outcome, it remains unclear whether poor DRQOL contributes to migraine burden, whether migraine-related symptoms impair dietary experiences, or whether these relationships are bidirectional; therefore, reverse causality cannot be ruled out. In addition, migraine status was assessed using a screening instrument rather than a formal clinical diagnosis of migraine or vestibular migraine. Consequently, the ROC analyses should be interpreted as evaluating discrimination of migraine screener positivity rather than diagnostic performance for migraine itself, and the identified cutoff value should be regarded as exploratory pending external validation.

Second, the inclusion of patients with psychogenic vertigo may have influenced the observed associations between DRQOL and psychological distress. To address this concern, we performed a sensitivity analysis excluding patients with psychogenic dizziness. The associations between DRQOL and SDS, HADS-A, HADS-D, and OD checklist scores remained significant and were generally comparable to those observed in the overall cohort. The persistence of these associations after exclusion of patients with psychogenic dizziness suggests that the observed relationships between DRQOL, psychological symptoms, and autonomic symptoms were not solely driven by the psychogenic dizziness subgroup. Nevertheless, the possibility of residual confounding cannot be excluded, and further studies focusing on more homogeneous diagnostic populations are warranted.

Third, the absence of objective nutritional assessment, such as dietary recall, food frequency questionnaires, food records, or nutritional biomarkers, limits the ability to determine whether DRQOL scores reflect actual dietary behaviors, dietary quality, or nutritional status, rather than primarily subjective and psychological perceptions of eating. This limitation is particularly relevant because the present study focused on DRQOL rather than objective dietary intake itself. In addition, objective autonomic assessments, such as heart rate variability, orthostatic blood pressure monitoring, or tilt-table testing, were not performed, limiting biological interpretation of the OD checklist findings. Because all key measures were self-reported, common-method bias may have inflated observed associations. This is particularly relevant for the observed correlations between DRQOL and psychological distress measures, as both constructs were assessed using similar subjective rating formats administered at the same time point, potentially exaggerating the strength of these relationships. The absence of a healthy control group further precludes assessment of whether elevated DRQOL scores are specific to dizziness-related disorders or instead reflect more general psychological distress among individuals seeking medical care. Future studies incorporating both DRQOL measures and objective nutritional and autonomic assessments will be necessary to clarify the relationship between dietary experiences, symptom burden, and actual biological or behavioral measures in patients with dizziness and balance disorders.

Finally, while the use of questionnaire-based symptom scores rather than diagnosis-based classifications limits diagnosis-specific conclusions, this approach is consistent with patient-centered outcome research and enables examination of symptom interrelationships across a broad severity spectrum, including subthreshold presentations that meaningfully affect daily functioning. An additional limitation is that it cannot be excluded that DRQOL primarily reflects psychological distress rather than diet-specific experiences, given its substantial correlations with anxiety and depression measures and the loss of independent association in adjusted models. Further studies are required to establish the discriminant validity of DRQOL relative to psychological distress constructs.

Despite these limitations, the present findings underscore the potential clinical relevance of DRQOL in patients with dizziness and migraine. DRQOL was associated with psychological distress and autonomic symptom burden, suggesting that DRQOL may reflect broader dimensions of patient well-being not captured by conventional vestibular assessments. Given these cross-sectional associations, future longitudinal and interventional studies are warranted to determine whether interventions targeting psychological distress, autonomic symptoms, or dietary experiences can improve DRQOL and related clinical outcomes.

### 4.6. Future Perspectives

Future studies should employ longitudinal designs to clarify the causal relationships among DRQOL, psychological distress, autonomic dysfunction, migraine, and vestibular symptoms. The incorporation of objective dietary measures, nutritional biomarkers, and autonomic assessments will help determine the biological relevance of DRQOL. Research exploring gut–brain–vestibular interactions, neuroinflammation, and psychosomatic mechanisms may further elucidate the pathways underlying these associations. Additionally, intervention studies evaluating nutritional, psychological, and multidisciplinary approaches are warranted to determine whether improving DRQOL can enhance clinical outcomes and overall QOL in patients with dizziness and balance disorders. Ultimately, larger multicenter studies are needed to validate the clinical applicability of DRQOL in vestibular practice.

## 5. Conclusions

This study demonstrates that DRQOL in patients with dizziness and balance disorders is associated with psychological distress and autonomic symptom burden and modestly discriminates between migraine screener-positive and migraine screener-negative patients. While DRQOL was not significantly correlated with individual migraine severity indices such as headache frequency or headache intensity, its assessment may contribute to a broader understanding of patients’ psychosomatic symptom burden and support patient-centered clinical evaluation that considers dietary experience alongside vestibular, autonomic, and psychological factors.

## Figures and Tables

**Table 1:** Demographic and clinical characteristics stratified by migraine screener status. Values are presented as mean ± standard deviation for continuous variables and number (percentage) for categorical variables. Continuous variables were compared using the Mann–Whitney U test, and categorical variables were compared using Fisher’s exact test. *p*-values are presented as unadjusted and Holm-adjusted for multiple comparisons. The migraine screener was treated as a dichotomous variable (negative vs. positive). Abbreviations are defined in the Abbreviations section.

	Migraine Screener			
	Negative	Positive	Unadjusted *p*-Value	Adjusted *p*-Value
	*n* = 79	*n* = 43		
Sex = M (%)	45 (57.0)	11 (25.6)	0.002	0.005
Age	42.9 ± 12.3	36 ± 12.5	0.004	0.012
DRQOL	40 ± 14.3	47.8 ± 15.1	0.006	0.012
HADS-A	6.96 ± 4.55	10.5 ± 4.39	<0.001	<0.001
SDS	42.3 ± 10	49.4 ± 8.72	<0.001	0.003
HADS-D	5.52 ± 3.91	7.49 ± 4.59	0.014	0.021
DHI	28.7 ± 18.9	45.6 ± 22	<0.001	<0.001
MIDAS	1.16 ± 0.59	2.44 ± 1.33	<0.001	<0.001
OD	4.77 ± 2.51	7.7 ± 2.02	<0.001	<0.001
Headache days	7.82 ± 16	21.4 ± 25.3	<0.001	<0.001
POUNDing	1.06 ± 1.3	2.98 ± 1.18	<0.001	<0.001
Headache intensity	2.16 ± 2.46	5.49 ± 1.88	<0.001	<0.001

## Data Availability

The data that support the findings of this study are available from the corresponding author upon reasonable request with the permission of the research ethics committee.

## Abbreviations

The following abbreviations are used in this manuscript:

AUC	Area under the curve
BPPV	Benign paroxysmal positional vertigo
CGRP	Calcitonin gene-related peptide
CI	Confidence interval
DASH	Dietary Approaches to Stop Hypertension
DHI	Dizziness Handicap Inventory
DRQOL	Diet-related quality of life
EGQ-D	Esophago-Gastric surgery and Quality of Dietary Life
HADS	Hospital Anxiety and Depression Scale
HADS-A	Hospital Anxiety and Depression Scale—Anxiety subscale
HADS-D	Hospital Anxiety and Depression Scale—Depression subscale
IBV	Idiopathic bilateral vestibulopathy
MD	Ménière’s disease
MIDAS	Migraine Disability Assessment Scale
MIND	Mediterranean-DASH Intervention for Neurodegenerative Delay
NRS	Numeric rating scale
OD	Orthostatic dysregulation
POUNDing	Pulsating, duration of 4–72 h, Unilateral, Nausea, Disabling
QOL	Quality of life
ROC	Receiver operating characteristic
SD	Standard deviation
SDS	Self-rating Depression Scale
VIF	Variance inflation factor

## Appendix A

**Algorithm A1.** Scripts used for analysis in R
library(readxl) library(corrplot) library(psych) library(Hmisc) tbl <- read_xls(“JRVNG20251104RT.xls”) sel <- tbl[,c(“Age”, “DHI”, “SDS”, “HADS/A”, “HADS/D”, “POUNDing”, “MIDAS”, “Days”, “Intensity”, “OD”, “DRQOL”)] colnames(sel) <- c(“Age”, “DHI”, “SDS”, “HADS-A”, “HADS-D”, “POUNDing”, “MIDAS”, “Headache days”, “Headache intensity”, “OD”, “DRQOL”) flattenCorrMatrix <- function(cormat, pmat) { ut <- upper.tri(cormat) data.frame( row = rownames(cormat)[row(cormat)[ut]], column = rownames(cormat)[col(cormat)[ut]], cor =(cormat)[ut], p = (pmat)[ut] ) } library(diptest) dip.test(sel$DRQOL) # Figure 2 # The p-values adjusted for multiple comparisons are displayed in the upper-right corner of the output results r1b <- corr.test(na.omit(sel), method = “spearman”, adjust = “holm”) A <- r1b$p A[lower.tri(A)] = 0 A = A+t(A) r1b$p <- A win.metafile(file=“corrplot-F2.emf”,width=12,height=12) corrplot(r1b$r, type=“upper”, order=“hclust”, method = ‘circle’, p.mat = r1b$p, sig.level = 0.001, insig = “blank”, tl.col = “black”, diag = FALSE, number.cex=1.5)$corrPos -> p1 text(p1$x, p1$y, round(p1$corr, 2)) dev.off() options(scipen=10) options(digits=3) flattenCorrMatrix(r1b$r, r1b$p) spearman_boot <- function(data, x, y, R=1000){ dat <- na.omit(data[, c(x,y)]) boot.fun <- function(d, i){ cor( d[i,1], d[i,2], method=“spearman” ) } b <- boot(dat, boot.fun, R=R) ci <- boot.ci( b, type=“perc” ) data.frame( Variable1 = x, Variable2 = y, rho = cor(dat[,1], dat[,2], method=“spearman”), Lower95CI = ci$percent [4], Upper95CI = ci$percent [5] ) } sig.cor <- subset( flattenCorrMatrix(r1b$r, r1b$p), p < 0.05 ) library(boot) CI.list <- lapply( 1:nrow(sig.cor), function(i){ spearman_boot(sel, sig.cor$row[i], sig.cor$column[i]) } ) CI.result <- do.call( rbind, lapply(CI.list, as.matrix) ) print(CI.result) # ======================================== # Exclusion of psychogenic causes sel <- tbl[,c(“Sex”, “Age”, “Diagnoses”, “DHI”, “SDS”, “HADS/A”, “HADS/D”, “POUNDing”, “MIDAS”, “Days”, “Intensity”, “OD”, “DRQOL”)] tsel = na.omit(sel) library(dplyr) library(tidyr) print( tsel %>% group_by(Diagnoses) %>% tally() ) tsel_e <- subset(tsel, !(tsel$`Diagnoses` == “Psychogenic”)) tsel_e <- tsel_e[,c(“Age”, “DHI”, “SDS”, “HADS/A”, “HADS/D”, “POUNDing”, “MIDAS”, “Days”, “Intensity”, “OD”, “DRQOL”)] colnames(tsel_e) <- c(“Age”, “DHI”, “SDS”, “HADS-A”, “HADS-D”, “POUNDing”, “MIDAS”, “Headache days”, “Headache intensity”, “OD”, “DRQOL”) r1b <- corr.test(na.omit(tsel_e), method = “spearman”, adjust = “holm”) A <- r1b$p A[lower.tri(A)] = 0 A = A+t(A) r1b$p <- A options(scipen=10) options(digits=3) res <- flattenCorrMatrix(r1b$r, r1b$p) print(subset(res, p < 0.05)) # Figure 1 library(devtools) library(ggplot2) library(Rmisc) sel <- tbl[,c(“DHI”, “SDS”, “HADS/A”, “HADS/D”, “POUNDing”, “MIDAS”, “Days”, “Intensity”, “OD”, “DRQOL”, “Age”)] tsel = na.omit(sel) options(digits=2) print(colMeans(tsel)) print(apply(tsel,2,sd)) col_blue <- “#5DADE2” # (DHI) col_green <- “#58D68D” # (SDS, HADS) col_orange <- “#F39C12” # (POUNDing, MIDAS, Headache) col_pink <- “#F1948A” # (OD) col_red <- “#E74C3C” # (DRQOL) col_purple <- “#AF7AC5” # (Age) p1 = ggplot(tsel, aes(x = DHI)) + geom_histogram(boundary = 0,binwidth=5,color=‘black’,fill=col_blue, closed =‘left’) + theme_light() + xlab(“DHI”) + ylab(““) + xlim(0,105) # 100 p2 = ggplot(tsel, aes(x = SDS)) + geom_histogram(boundary = 0,binwidth=5,color=‘black’,fill=col_green, closed =‘left’) + theme_light() + xlab(“SDS”) + ylab(““) + xlim(20,85) # 20-80 p3 = ggplot(tsel, aes(x = `HADS/A`)) + geom_histogram(boundary = 0,binwidth=1,color=‘black’,fill=col_green, closed =‘left’) + theme_light() + xlab(“HADS-A”) + ylab(““) + xlim(0,22) # 0-21 p4 = ggplot(tsel, aes(x = `HADS/D`)) + geom_histogram(boundary = 0,binwidth=1,color=‘black’,fill=col_green, closed =‘left’) + theme_light() + xlab(“HADS-D”) + ylab(““) + xlim(0,22) # 0-21 p5 = ggplot(tsel, aes(x = POUNDing)) + geom_histogram(boundary = 0,binwidth=1,color=‘black’,fill=col_orange,closed =‘left’) + theme_light() + xlab(“POUNDing”) + ylab(““) + xlim(0,6) # 0-5 p6 = ggplot(tsel, aes(x = MIDAS)) + geom_histogram(boundary = 0,binwidth=1,color=‘black’,fill=col_orange,closed =‘left’) + theme_light() + xlab(“MIDAS”) + ylab(““) + xlim(1,5) # 1-4 p7 = ggplot(tsel, aes(x = Days)) + geom_histogram(boundary = 0,binwidth=5,color=‘black’,fill=col_orange,closed =‘left’) + theme_light() + xlab(“Headache days”) + ylab(““) + xlim(0,95) # 0-90 p8 = ggplot(tsel, aes(x = Intensity)) + geom_histogram(boundary = 0,binwidth=1,color=‘black’,fill=col_orange,closed =‘left’) + theme_light() + xlab(“Headache intensity”) + ylab(““) + xlim(0,11) # 0-10 p9 = ggplot(tsel, aes(x = OD)) + geom_histogram(boundary = 0,binwidth=1,color=‘black’,fill=col_pink, closed =‘left’) + theme_light() + xlab(“OD”) + ylab(““) + xlim(0,13) # 0-11 p0 = ggplot(tsel, aes(x = DRQOL)) + geom_histogram(boundary = 0,binwidth=3,color=‘black’,fill=col_red, closed =‘left’) + theme_light() + xlab(“DRQOL”) + ylab(““) + xlim(18,93) # 18-90 (72) pa = ggplot(tsel, aes(x = Age)) + geom_histogram(boundary = 0,binwidth=5,color=‘black’,fill=col_purple,closed =‘left’) + theme_light() + xlab(“Age”) + ylab(““) + xlim(10,70) # 14-65 win.metafile(file=“hist-F1.emf”,width=10,height=15) layout <- matrix(c(1, 2, 3, 4, 5, 6, 7, 8, 9, 10, 11, 12), nrow = 6, byrow = TRUE) multiplot(plotlist=list(p1,p2,p3,p4,p5,p6,p7,p8,p9,p0,pa,NULL), layout = layout) dev.off() sel <- tbl[,c(“Sex”, “Age”, “DHI”, “SDS”, “HADS/A”, “HADS/D”, “POUNDing”, “MIDAS”, “Days”, “Intensity”, “OD”, “DRQOL”, “Screener”)] colnames(sel) <- c(“Sex”, “Age”, “DHI”, “SDS”, “HADS-A”, “HADS-D”, “POUNDing”, “MIDAS”, “Headache days”, “Headache intensity”, “OD”, “DRQOL”, “Migraine screener”) tsel = na.omit(sel) tsel$`Migraine screener` <- as.factor(tsel$`Migraine screener`) library(tableone) table <- CreateTableOne(strata=“Migraine screener”, data=tsel, test=T, testNonNormal = kruskal.test, testExact = fisher.test) print(table) summary(table) pvals <- attr(table$ContTable, “pValues”) df <- data.frame( Variable = rownames(pvals), pNonNormal = pvals$pNonNormal, pAdj_Holm = p.adjust(pvals$pNonNormal, method = “holm”) ) print(df) pvals <- attr(table$CatTable, “pValues”) df <- data.frame( Variable = rownames(pvals), pExact = pvals$pExact, pAdj_Holm = p.adjust(pvals$pExact, method = “holm”) ) print(df) # ======================================== library(epiDisplay) library(pROC) library(car) GLM.1 <- glm(data=tsel, `Migraine screener` ~ DRQOL, family = binomial(link = “logit”)) summary(GLM.1) options(digits=4) print(exp(coef(GLM.1))) print(exp(confint(GLM.1))) GLM.2 <- glm(data=tsel, `Migraine screener` ~ DRQOL + Sex + Age, family = binomial(link = “logit”)) summary(GLM.2) options(digits=4) print(exp(coef(GLM.2))) print(exp(confint(GLM.2))) print(vif(GLM.2)) ROC.1 <- roc(response=tsel$`Migraine screener`, predictor=predict(GLM.1, type=“response”)) # plot(1-ROC.1$specificities, ROC.1$sensitivities, xlab=“1-Specificity”, ylab=“Sensitivity”, type=“l”, lwd=2) # abline(a=0,b=1,lty=2) CI.1<-ci.auc(ROC.1, conf.level=0.95) cutoff.1 <- coords(ROC.1, x=“best”, ret=c(“threshold”, “sensitivity”, “specificity”, “ppv”, “npv”), best.method=“youden”) c.point.1 <- cutoff.1[1] beta.1 <- coef(GLM.1) # The regression coefficients (log odds ratios) of the logistic model cutoff.1.variable <- (log(c.point.1/(1-c.point.1)) - beta.1[“(Intercept)”]) / beta.1[“DRQOL”] # Cutoff value for the original variable print(ROC.1$auc) print(CI.1) # 95% confidence interval for the area under the curve (AUC) print(c.point.1) # Cutoff values in regression analysis models print(cutoff.1.variable) # Optimal cutoff value when there is only one explanatory variable (ignore if there are multiple explanatory variables) print(cutoff.1[2:5]) # sensitivity / specificity / PPV (positive predictive value) / NPV (negative predictive value) ROC.2 <- roc(response=tsel$`Migraine screener`, predictor=predict(GLM.2, type=“response”)) CI.2<-ci.auc(ROC.2, conf.level=0.95) print(ROC.2$auc) print(CI.2) # compare_auc <- function(formula1, formula2, data, response, alternative=“two.sided”){ olm1 <- glm(formula1, data=data, family=“binomial”) olm2 <- glm(formula2, data=data, family=“binomial”) ROC_1 <- pROC::roc(response=response, predictor=fitted(olm1)) ROC_2 <- pROC::roc(response=response, predictor=fitted(olm2)) print(pROC::roc.test(ROC_1, ROC_2, method=“delong”, alternative=alternative)) print(pROC::roc.test(ROC_1, ROC_2, method=“bootstrap”, boot.n = 1000, alternative=alternative)) } compare_auc(`Migraine screener` ~ DRQOL,`Migraine screener` ~ DRQOL + Sex + Age,tsel,tsel$`Migraine screener`,”two.sided”) GLM.5 <- glm(data=tsel, `Migraine screener` ~ DRQOL + Sex + Age + `HADS-A` + `HADS-D` + SDS + OD, family=binomial(link = “logit”)) summary(GLM.5) options(digits=4) print(coef(GLM.5)) print(exp(coef(GLM.5))) print(exp(confint(GLM.5))) print(vif(GLM.5)) ROC.5 <- roc(response=tsel$`Migraine screener`, predictor=predict(GLM.5, type=“response”)) CI.5<-ci.auc(ROC.5, conf.level=0.95) print(ROC.5$auc) print(CI.5)

## References

[1] Katzenberger B., Fuchs S., Schwettmann L., Strobl R., Hauser A., Koller D., Grill E. (2023). Association of self-efficacy, risk attitudes, and time preferences with functioning in older patients with vertigo, dizziness, and balance disorders in a tertiary care setting—Results from the MobilE-TRA2 cohort. Front. Neurol..

[2] Lindell E., Odhagen E., Tuomi L. (2024). Living with dizziness impacts health-related quality of life among older adults. Laryngoscope Investig. Otolaryngol..

[3] Hac N.E.F., Gold D.R. (2024). Advances in diagnosis and treatment of vestibular migraine and the vestibular disorders it mimics. Neurotherapeutics.

[4] Villar-Martinez M.D., Goadsby P.J. (2024). Vestibular migraine: An update. Curr. Opin. Neurol..

[5] Gazerani P. (2020). Migraine and diet. Nutrients.

[6] Balali A., Karimi E., Kazemi M., Hadi A., Askari G., Khorvash F., Arab A. (2024). Associations between diet quality and migraine headaches: A cross-sectional study. Nutr. Neurosci..

[7] Nguyen K.V., Schytz H.W. (2024). The Evidence for Diet as a Treatment in Migraine—A Review. Nutrients.

[8] Andersen B.V., Chan R.C.K., Byrne D.V. (2021). A conceptual framework for multi-dimensional measurements of food related pleasure—The food pleasure scale. Foods.

[9] Jaeger S.R., Vidal L., Chheang S.L., Ares G. (2022). Consumer conceptualisations of food-related wellbeing: An exploration of wellbeing-related terms in four industrialised countries. Appetite.

[10] Jaeger S.R., Vidal L., Chheang S.L., Ares G. (2023). Dimensions of food-related wellbeing and their relative importance among New Zealand consumers: A quasi-replication and extension approach. Appetite.

[11] Alfawaz W., Albassam R.S., Almuharib N., Alghafis S., Mahfouz W. (2025). Association between diet and quality of life among healthcare professionals in King Saud University Medical City. Front. Public Health.

[12] Fitzgerald K.C., Tyry T., Salter A., Cofield S.S., Cutter G., Fox R., Marrie R.A. (2018). Diet quality is associated with disability and symptom severity in multiple sclerosis. Neurology.

[13] Chen Z., Zhang H., Jin J., Su C., Chen H., Li B. (2025). A longitudinal study of dietary inflammatory index and quality of life in people with osteoarthritis: Data from the Osteoarthritis Initiative database. Sci. Rep..

[14] Tamber A.L., Wilhelmsen K.T., Strand L.I. (2009). Measurement properties of the Dizziness Handicap Inventory by cross-sectional and longitudinal designs. Health Qual. Life Outcomes.

[15] Koppelaar-Van Eijsden H.M., Schermer T.R., Bruintjes T.D. (2022). Measurement Properties of the Dizziness Handicap Inventory: A Systematic Review. Otol. Neurotol..

[16] Honda K., Tanaka K., Tanimura M. (1997). Clinical manifestation, criteria and reproducibility of orthostatic hypotension. Modern Orthostatic Hypotension.

[17] Masood M.A., Asif N., Younus M., Singla B., Khan S., Singla S., Elahi S.H., Parveen N., Ishfaq M.A., Jamil T. (2025). Association Between Gut-Brain Axis Dysfunction and Vestibular Migraine Severity. Cureus.

[18] Palazzo C.C., Leghi B.E., Diez-Garcia R.W. (2022). Food Consciousness Intervention Improves Interoceptive Sensitivity and Expression of Exteroception in Women. Nutrients.

[19] Iwasa H., Yoshida Y., Suzukamo Y. (2019). Psychometric properties of the diet-related quality of life (DRQOL) scale and its short version among older adults. Nihon Koshu Eisei Zasshi Jpn. J. Public Health.

[20] Pemau R.C., González-Palacios P., Kerr K.W. (2024). How quality of life is measured in studies of nutritional intervention: A systematic review. Health Qual. Life Outcomes.

[21] Patelarou E., Giakoumidakis K. (2025). Nutrition and Quality of Life for Patients with Chronic Disease. Nutrients.

[22] Furman J.M., Balaban C.D., Jacob R.G., Marcus D.A. (2005). Migraine-anxiety related dizziness (MARD): A new disorder?. J. Neurol. Neurosurg. Psychiatry.

[23] Jacobson G.P., Newman C.W. (1990). The Development of the Dizziness Handicap Inventory. Arch. Otolaryngol. Neck Surg..

[24] Zigmond A.S., Snaith R.P. (1983). The Hospital Anxiety and Depression Scale. Acta Psychiatr. Scand..

[25] Zung W.W.K. (1965). A Self-Rating Depression Scale. Arch. Gen. Psychiatry.

[26] Honda K., Nose T., Yoshida N., Tanimura M., Tanaka K. (1977). Responses to the Postural Change and Orthostatic Dysregulation Symptoms: A Population Study on Japanese Junior and Senior High School Students. Jpn. Circ. J..

[27] Tanaka H., Fujita Y., Takenaka Y., Kajiwara S., Masutani S., Ishizaki Y., Matsushima R., Shiokawa H., Shiota M., Ishitani N. (2009). Japanese clinical guidelines for juvenile orthostatic dysregulation version 1. Pediatr. Int..

[28] Detsky M.E., McDonald D.R., Baerlocher M.O., Tomlinson G.A., McCrory D.C., Booth C.M. (2006). Does this patient with headache have a migraine or need neuroimaging?. JAMA.

[29] Stewart W.F., Lipton R.B., Dowson A.J., Sawyer J. (2001). Development and testing of the Migraine Disability Assessment (MIDAS) Questionnaire to assess headache-related disability. Neurology.

[30] Takeshima T., Sakai F., Suzuki N., Shimizu T., Igarashi H., Araki N., Manaka S., Nakashima K., Hashimoto Y., Iwata M. (2015). A simple migraine screening instrument; Validation study in Japan. Jpn. J. Headache.

[31] Suzukamo Y., Fukuhara S., Ono C. (2001). Diet and quality of life (QOL). Geriatr. Med..

[32] R Core Team (2025). R: A Language and Environment for Statistical Computing.

[33] Tu Y.H., Chang C.M., Yang C.C., Tsai I.J., Chou Y.C., Yang C.P. (2025). Dietary Patterns and Migraine: Insights and Impact. Nutrients.

[34] Liu X., Yu Y., Hou L., Yu Y., Wu Y., Wu S., He Y., Ge Y., Wei Y., Luo Q. (2023). Association between dietary habits and the risk of migraine: A Mendelian randomization study. Front. Nutr..

[35] Amani Tirani S., Askari G., Khorvash F., As’habi A., Arab A. (2023). Associations between dietary diversity score and migraine headaches: The results from a cross-sectional study. Front. Nutr..

[36] Legesse S.M., Addila A.E., Jena B.H., Jikamo B., Abdissa Z.D., Hailemarim T. (2025). Irregular meal and migraine headache: A scoping review. BMC Nutr..

[37] Behrouz V., Hakimi E., Mir E. (2025). Impact of Dietary Patterns on Migraine Management: Mechanisms of Action and Recent Literature Insights. Brain Behav..

[38] Dacka M., Sobczyk M., Dąbrowska P., Giżewska K., Żuber M. (2024). Role of calcitonin gene-related peptide (CGRP) receptor antagonist in acute and preventive treatment of migraine. Prospect. Pharm. Sci..

[39] Kupczyk D., Bilski R., Szeleszczuk Ł., Mądra-Gackowska K., Studzińska R. (2025). The Role of Diet in Modulating Inflammation and Oxidative Stress in Rheumatoid Arthritis, Ankylosing Spondylitis, and Psoriatic Arthritis. Nutrients.

[40] Fedorowski A. (2019). Postural orthostatic tachycardia syndrome: Clinical presentation, aetiology and management. J. Intern. Med..

[41] Benrud-Larson L.M., Dewar M.S., Sandroni P., Rummans T.A., Haythornthwaite J.A., Low P.A. (2002). Quality of life in patients with postural tachycardia syndrome. Mayo Clin. Proc..

[42] Molero P., De Lorenzi F., Gędek A., Strater C., Popescu E., Ortuño F., Van Der Does W., Martínez-González M.A., Molendijk M.L. (2025). Diet quality and depression risk: A systematic review and meta-analysis of prospective studies. J. Affect. Disord..

[43] Machado P., Livingstone K.M., Denniss E., Marchese L.E., Lawrence M.A., McNaughton S.A. (2026). Development and evaluation of a multidimensional diet quality score for sustainable healthy diets (SUSDIET). Appetite.

[44] Robinson E., Blissett J., Higgs S. (2012). Changing memory of food enjoyment to increase food liking, choice and intake. Br. J. Nutr..

[45] Kronrod A., Hammar M.E., Lee J.S., Thind H.K., Mangano K.M. (2021). Linguistic Delight Promotes Eating Right: Figurative Language Increases Perceived Enjoyment and Encourages Healthier Food Choices. Health Commun..

[46] Honda M., Wakita T., Onishi Y., Nunobe S., Miura A., Nishigori T., Kusanagi H., Yamamoto T., Boddy A., Fukuhara S. (2015). Development and Validation of a Disease-Specific Instrument to Measure Diet-Targeted Quality of Life for Postoperative Patients with Esophagogastric Cancer. Ann. Surg. Oncol..

[47] Cornil Y., Chandon P. (2016). Pleasure as an ally of healthy eating? Contrasting visceral and Epicurean eating pleasure and their association with portion size preferences and wellbeing. Appetite.

